# Job characteristics, marital intentions, and partner-seeking actions: Longitudinal evidence from Japan

**DOI:** 10.4054/demres.2020.43.52

**Published:** 2020-12-09

**Authors:** Wei-hsin Yu, Yuko Hara

**Affiliations:** 1 University of California, Los Angeles.; 2 University of Maryland, College Park.

## Abstract

**BACKGROUND:**

Most research linking jobs to marriage formation focuses on how job contexts and prospects affect singles’ paces of entering marriage. Direct evidence on whether job traits shape singles’ desire for marriage and actions toward forming a union remains scarce.

**OBJECTIVE:**

We examine how changes in a range of job characteristics correspond to alterations in never-married people’s intention to marry and actions taken to meet romantic partners in Japan, a country with increasing inequality in job quality and declining marriage rates.

**METHODS:**

We use longitudinal data from the Japan Life Course Panel Survey to fit fixed-effects models, which take into account unobserved heterogeneity among people with differing jobs.

**RESULTS:**

We find that rises in job insecurity and workplace staffing shortages weaken, whereas increases in income and job autonomy strengthen, men’s intention to marry. Moreover, men with a low marriage desire are especially likely to withdraw from partner-seeking activities when they have low-income jobs or face great deadline pressure at work. Job prospects and quality are generally less important to women’s desire for marriage or partner-seeking actions. Nevertheless, being in workplaces where teamwork is prevalent, which could enhance singles’ exposure to married and older coworkers, raises both women’s intention to marry and their probability of using a formal method, such as employing a marriage agency, to find a partner.

**CONCLUSIONS:**

For Japanese men, our results offer support for the argument that economic stagnation and deterioration of job quality are conducive to later and fewer marriages. The findings for women, however, are more consistent with the narrative focusing on values and social influences.

**CONTRIBUTIONS:**

This study enriches our understanding of singles’ considerations of marriage and partner search and provides highly rigorous evidence on the roles of job conditions.

## 1. Introduction

Demographers and family researchers have observed the trend toward later and fewer marriages across industrialized countries (Haskey 1995; Jones 2007; Lesthaeghe 2010; Martin, Astone, and Peters 2014). Despite its common occurrence, this trend does not bear the same consequences for all societies. In the United States, for example, even though people are marrying at increasingly older ages, the rises in cohabitation and nonmarital childbearing have substantially offset the impacts of marriage delay on individuals’ chances of entering romantic unions and parenthood (Edin and Kefalas 2005; Manning, Brown, and Payne 2014). Conversely, marriage declines drastically reduce union experiences in East Asia, where cohabitation rarely serves as an alternative to marriage (Raymo, Iwasawa, and Bumpass 2009; Raymo et al. 2015). The steady marriage delay also leads to exceptionally low fertility rates in that region (Jones 2007; Raymo et al. 2015), where childbirths outside of marriage remain stigmatized and few (Hertog and Iwasawa 2011).

Much research has been devoted to understanding the decline and postponement of marriage, especially in East Asia, given the consequences of marriage delay there (Raymo et al. 2015). Such research tends to analyze the pace of entering marriage (Ohlsson-Wijk 2011; Oppenheimer, Kalmijn, and Lim 1997; Raymo 2003; Sweeney 2002; Xie et al. 2003; Yu and Kuo 2016; Yu, Su, and Chiu 2012). Focusing on the speed of marriage formation, however, likely confounds singles’ intention to marry, as well as their willingness to devote time and energy to finding marriage partners, with factors beyond their control. A shortage of available partners and a poor reception in the marriage market, for example, could decelerate singles’ marriage transitions as does their lack of desire for marriage. Meanwhile, explanations of marriage delay, such as stressing how women’s rising economic opportunities reduce their incentives to marry (Raymo et al. 2015: 480), often imply that the relevant factors shape marriage timing by dampening one’s intention to marry. Few studies, however, examine singles’ marital intentions – except for some concerning cohabiting couples’ marital intentions (e.g., Guzzo 2009; Kuo and Raley 2016a; Vespa 2014) – and even fewer address how singles differ in their tendencies to take actions to find marriage partners.

In addition to lacking analyses of singles’ marital intentions and partner-seeking actions, prior research offers insufficient insights on how various job traits are linked to these intentions and actions. Many explanations for marriage delay would logically expect job attributes to play a role. For example, the argument that having greater economic prospects weakens women’s marital intentions implies that women whose jobs promise more promotion opportunities or greater security should express a lower marital intention and take fewer actions to seek partners. Similarly, if a sense of financial insecurity in uncertain economic times is thought to raise men’s fear of inability to fulfill the family-provider role, thereby decreasing their interest in marriage (Raymo et al. 2015: 482), we should find men whose workplaces signal little stability to desire marriage less. Although some researchers have investigated the link between nonstandard employment status and marriage timing (Piotrowski, Kalleberg, and Rindfuss 2015), a comprehensive analysis addressing various job characteristics remains rare.

This study uses longitudinal data from Japan to examine how differing job conditions, including the job’s likelihoods to impose deadlines, to require long hours, to facilitate workers’ skill growth, to enable control over the tasks, and to be terminated in the near future, are tied to changes in marital intention status for singles who have never been married (‘singles’ hereafter for simplicity) over time. We define a marital intention as an expressed desire for marriage and use the intention and desire to marry interchangeably throughout the paper. To move beyond the limited research on job traits and marital intentions (Yu and Kuo 2017), we also investigate how marital intention status and job characteristics jointly contribute to alterations in singles’ partner-seeking behavior. In particular, we ask whether job attributes may affect how likely single people will act on their marital intentions. That is, our analysis addresses how different job characteristics may amplify or weaken the relationship between marital intentions and partner-seeking behavior.

Because Japan exemplifies a country with very low fertility and few alternatives to marriage and marital births (Raymo, Iwasawa, and Bumpass 2009; Raymo et al. 2015), studying marital intentions and partner search in this country can enhance our understanding of the underlying causes for demographic changes in similar contexts, such as other East Asian societies. Furthermore, Japan is ideal for examining the influences of job attributes. With a gender context in which marriage brings disproportionate domestic obligations for women and considerable financial burden for men (Nemoto, Fuwa, and Ishiguro 2013; Ono 2003), Japanese singles’ considerations about marriage and partner seeking are especially likely to be intertwined with their job conditions, which can facilitate or inhibit the new roles they expect to face with marriage. Finally, by analyzing job attributes’ relevance to singles’ marital intentions and partner search in Japan, a society that has experienced steady rises in precarious employment (Raymo and Shibata 2017; Yu 2012), we also gain knowledge about how work intersects with family plans in contexts undergoing similar changes.

## 2. Explanations for marriage decline and the relevance of jobs

Scholars aiming to explain the trend toward later and fewer marriages often focus on changes in relative benefits and costs of marriage (Bumpass et al. 2009), in individuals’ ability to meet the expectations surrounding marriage (Edin and Kefalas 2005; Gibson-Davis, Edin, and McLanahan 2005; Raymo and Shibata 2017), or in the societal views on the institution of marriage (Retherford, Ogawa, and Matsukura 2001; Retherford, Ogawa, and Sakamoto 1996). In either scenario, job attributes can be expected to shape the extent to which singles intend to marry, because these attributes may affect their calculations of the potential gain or loss from marriage, their ability to afford the expected life styles for married people, and their chances of being exposed to more traditional family values in the workplace (Yu and Kuo 2017). Likewise, job attributes may be linked to the actions singles take to seek romantic partners, especially in Japan, where marriage is the presumed destination of romantic relationships. Below we discuss in detail the differing perspectives about marriage delay and decline, as well as how job traits may shape marital intentions and partner seeking according to each perspective. As the explanations for marriage trends are not necessarily mutually exclusive (Raymo et al. 2015; Retherford, Ogawa, and Matsukura 2001), the job characteristics related to differing explanations may also simultaneously account for variations in singles’ marriage desire and partner-seeking behavior.

### 2.1 Gender inequality and opportunity costs of marriage

One frequently cited explanation for the low fertility in industrialized countries is the persistence of gender inequality within the household. This inequality, alongside with rises in women’s employment opportunities, leads women to anticipate heightened work-family conflict with childbearing; women are hence reluctant to become mothers (McDonald 2000; Morgan and Taylor 2006). This perspective can be extended to explain the trend toward later and fewer marriages in Japan. Because childbearing is closely tied to marriage and even considered as the primary reason for marriage (Raymo and Iwasawa 2008; Raymo et al. 2015: 480), disincentives for Japanese women to become mothers could similarly dampen their intention to marry and eagerness to seek romantic partners.

More than their counterparts in other advanced economies, Japanese women may see disincentives for marriage and childrearing. Although gender inequality within the household is not unique to Japan, comparative research shows that Japanese husbands’ share of household work is exceptionally small (Geist 2005; Qian and Sayer 2016), with many of them spending no time on domestic chores at all (Tsuya, Bumpass, and Choe 2000; Tsuya et al. 2005). The root of Japanese men’s low participation in household work is the common acceptance of a rigid gender division of labor within marriage. Not only are there prevailing cultural expectations for husbands to provide and wives to care for their families, but many social institutions, such as the employment, school, and childcare systems, are also designed to reinforce the male-breadwinner family model in Japan (Hirao 2001; Yu 2009). As a result, both men and women often see gender specialization within marriage as inevitable, despite their discontent (Brinton et al. 2018).

While marriage has persistently brought Japanese women considerable domestic obligations, the steady rise in single women’s educational attainment has potentially opened up options outside of marriage (Bumpass et al. 2009). Even though the erosion of the gender gaps in occupational status and wages has been slow (Yu 2009), Japanese women are able to afford singlehood with meagerly paid jobs, given the custom of young adults living with parents until marriage (Raymo et al. 2015; Yu and Kuo 2016). With time, single women with jobs may increasingly see the sacrifices of autonomy and leisure time, which are considered as inevitable with marriage (Nomaguchi 2006), as not worthy of the potential economic gain from marriage.

If the growing awareness of the opportunity costs of marriage indeed drives Japanese women to lose interest in marriage, then we should find the intention to marry to be lower when women have more to lose with marriage. Job attributes that promise better prospects, such as a high wage and the provision of skill accumulation opportunities, may make the potential work-family conflict that marriage will impose less tolerable; these attributes may therefore be associated with a lower intention to marry and fewer actions taken to seek romantic partners. Conversely, job characteristics that reduce the appeal of paid work, such as low job autonomy, great pressure for meeting deadlines, the need to constantly deal with staff shortages, and job insecurity, may make marriage seem like a better alternative (Yu and Kuo 2017). Even if women do not plan to leave their jobs upon marriage, the reduced work effort compelled by the increase in domestic obligations with marriage would seem less consequential if they do not find their jobs worth keeping in the long run. Thus, based on the perspective of gender inequality and the opportunity costs of marriage, we can expect that women with promising job traits will express a lower intention to marry and engage in fewer partner-seeking activities, whereas those with undesirable traits will do the opposite.

### 2.2 Economic instability and precarious employment conditions

A separate argument for the decline in marriage emphasizes rising economic instability and deteriorations in working conditions. Studies of marriage timing in the United States have long considered economic circumstances to be important to individuals’ decision to marry (Oppenheimer 1988). Because the social expectations for marriage include that the marrying couple is capable of establishing an independent household that meets a certain living standard (Gibson-Davis, Edin, and McLanahan 2005), and because men are supposed to provide for the family, men who have reached more stable career stages and have higher income potential are found to transition to marriage faster (Oppenheimer, Kalmijn, and Lim 1997; Xie et al. 2003). With rising prevalence of women’s employment, the importance of their financial contribution to the family has also grown in the United States, making marriage rates increasingly positively associated with women’s economic prospects (Sweeney 2002). Confirming the link between US women’s economic conditions and marriage considerations, ethnographic research further shows that low-income women tend to perceive a discrepancy between their economic status and the financial stability customarily portrayed for married couples, resulting in their avoidance of marriage (Edin and Kefalas 2005).

Based on the US research about economic circumstances, some propose that the macroeconomic shifts that raise the overall sense of economic instability, or, at least, increase the share of singles who find marriage and parenthood unaffordable, also explain the marriage trends in East Asia (Raymo et al. 2015). Japan, in particular, has experienced prolonged economic stagnation from the 1990s to 2000s, which contrasted sharply with the previous decades, featuring rapid economic growth and long-term employment (for men). Although the economic stagnation may not have fundamentally transformed Japan’s employment system (Yu 2010), it has led to considerable increases in management’s use of temporary, contract-based workers, who tend to be poorly paid, deprived of job security and fringe benefits, and facing low odds of upward mobility (Yu 2012). Even for those not in precarious employment conditions, Japan’s long stagnation has largely diminished the norm from the previous era, that virtually all working-age men can expect to be employed and given a wage sufficient to support the entire family until retirement (Yu 2009). Given the strong expectation for Japanese men to provide for their wives and children, such changes are especially likely to provoke anxiety among men, making them increasingly skeptical about the affordability of marriage and the subsequent phase, parenthood.

Needless to say, the difficulty to afford marriage is not evenly distributed among Japanese men. Research shows that those with nonstandard employment status enter marriage at a slower pace (Piotrowski, Kalleberg, and Rindfuss 2015). Rises in men’s unemployment rates are also found to contribute to fewer marriages, suggesting that jobless men are less likely to marry (Raymo and Shibata 2017). Although part of the reason why men with precarious employment status experience late marriage may be that they are deemed “unmarriageable” by potential partners, some ethnographic research suggests that the anxiety over providing for the family does decrease men’s interest in marriage (Nemoto, Fuwa, and Ishiguro 2013). A systematic analysis on how changes in specific job conditions, rather than just employment status, are responsible for alterations in men’s marital intentions is nevertheless needed. If economic concerns are indeed relevant for Japanese men, we should find they desire marriage more when their jobs are highly paid, relatively secure, and in economically stable workplaces (e.g., no constant staff shortages).

As for Japanese women, some research indicates that those with low wages or nonstandard jobs began to enter marriage more slowly in recent years (Fukuda 2013; Piotrowski, Kalleberg, and Rindfuss 2015). Marriage timing, however, does not necessarily reflect marital intentions. Job attributes that signal perilous economic conditions still may not dampen Japanese women’s intention to marry as they may do men’s. With the evidence that rises in women’s unemployment and nonstandard employment have increased fertility, Raymo and Shibata (2017) argue that Japan’s gender context makes women less likely to consider their own economic stability as a precondition for marriage and childbearing. Yu and Kuo (2017) also find job attributes related to economic prospects barely affect women’s intention to marry, although their reliance on cross-sectional data, which cannot address unobserved heterogeneity, constitutes a weakness.

**Figure 1:**
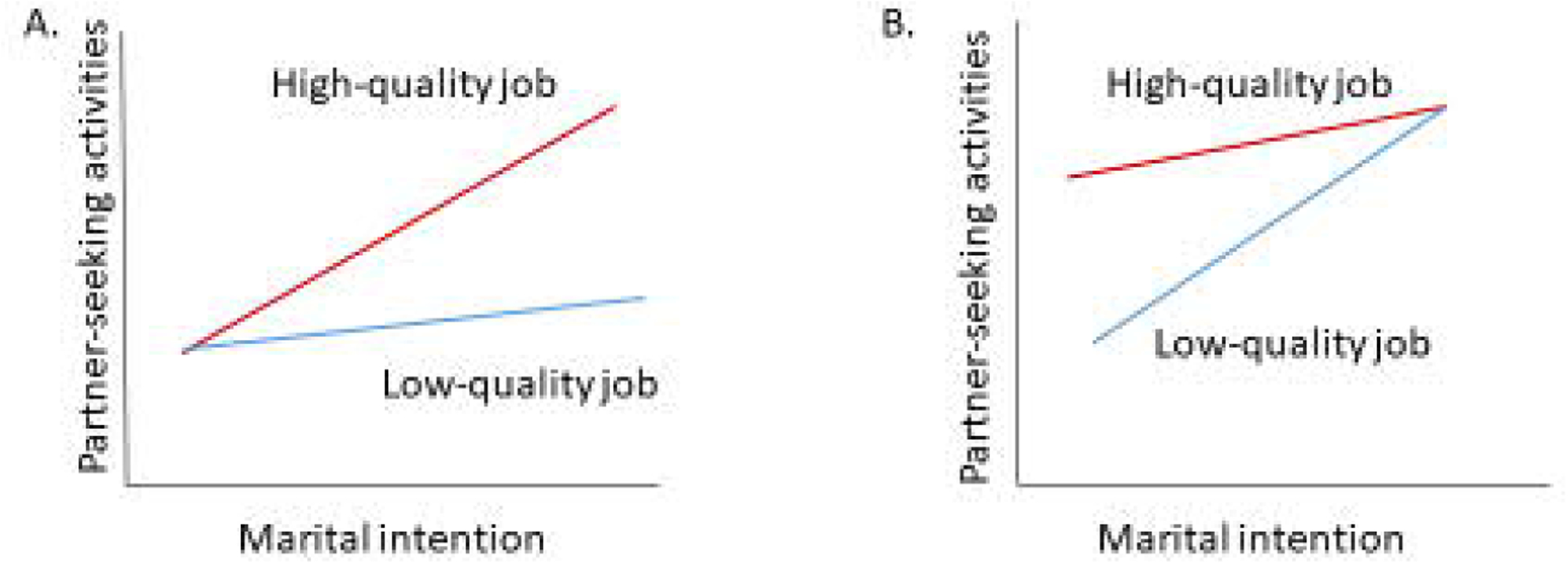
Possible relationships among job quality, marital intention, and partner seeking

Beyond shaping the intention to marry, job conditions may also affect how singles with varying intention statuses would take actions to seek romantic partners. While partner-search behavior should somewhat reflect the desire for marriage – that is, those eager to marry should be more active in seeking partners – it is possible that those with similar intention statuses are not equally active in partner seeking, because their actions are confined by the different resources their jobs provide. Jobs that pay more or do not require excessive hours may enable individuals to spend more money and time on matchmaking services or social activities through which they can meet a partner. Jobs that allow limited autonomy or impose unduly stress from deadlines or staff shortages may overwhelm individuals to the extent that they have little capacity for developing personal life (Kuo and Raley 2016b). Such job traits may therefore deter partner search even when individuals clearly desire marriage. Panel A in Figure 1 illustrates the hypothetical relationships between marital intention status and engagement in partner-seeking activities for those with high- and low-quality jobs, with the assumption that high-quality jobs help realize a strong marital intention.

**Figure 2:**
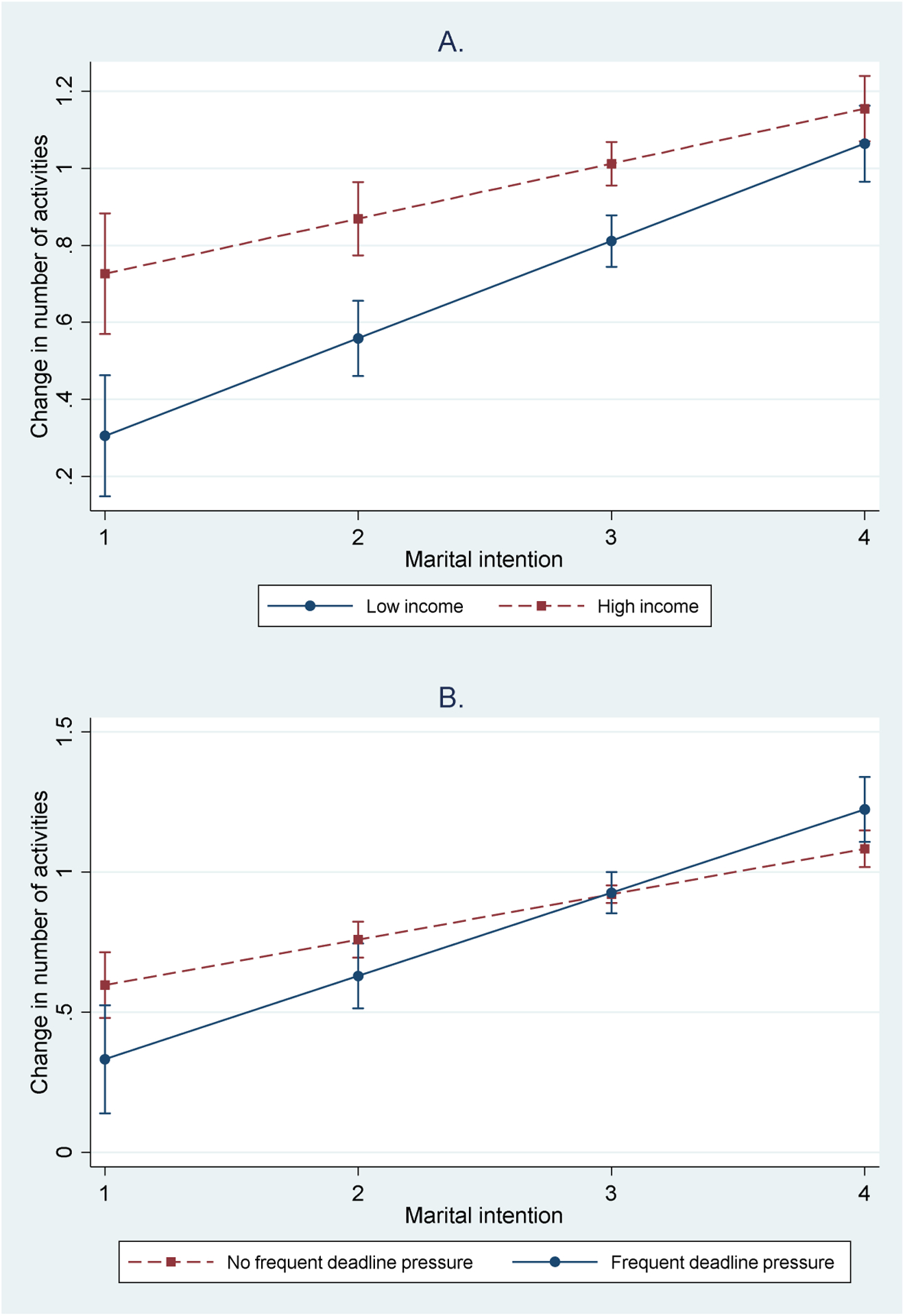
Predicted numbers of partner-seeking activities by marital intention and job attributes for men *Note*: The predicted probabilities are calculated using coefficients in Model 3 for men in Table 3. The predicted values are presented with 95% confidence intervals. Because overlapped confidence intervals do not indicate no difference between the predicted values, we also test whether the difference between each pair of predicted values is statistically different from zero. In most cases (except for when the pair of predicted values virtually overlap) the p-value for the test is smaller than 0.05.

Alternatively, it is possible that job characteristics hamper or facilitate partner search more for singles who are lukewarm about marriage, if those eager to marry will find time and resources to be engaged in partner-seeking activities regardless of their job quality. Panel B in Figure 2 illustrates this scenario, where all those desiring marriage strongly participate in partner-seeking activities to similar extents; having a high-quality job mainly encourages those less eager to marry to seek romantic partners more actively. Because having a romantic partner is the key to the transition to marriage in Japan (Yu and Kuo 2016), even if having high-quality jobs merely helps those ambivalent about marriage to be relatively active in partner search, this activeness may still accelerate marriage formation.

### 2.3 Ideology and exposure to values

A third explanation for demographic changes, including marriage and fertility declines, stresses the role of norms and values (Atoh 2001; Morgan and Taylor 2006). In Japan, the greater emphasis on the emotional component of marriage, the rising acceptance of premarital sex, and a modest decline in the ideal number of children are all thought to make singles less hasty about their transitions to marriage (Retherford, Ogawa, and Matsukura 2001; Retherford, Ogawa, and Sakamoto 1996). As incidents of late marriage increase, their social acceptance also grows, because people who know singles above the typical marriage age are more likely to approve of marriage postponement (Rindfuss et al. 2004). Through social networks and exposure, new norms about marriage and marriage timing diffuse and prevail, which further fuel family and demographic changes (Choe et al. 2014).

Several studies show that workplace interactions constitute an important means for individuals to be exposed to others’ family values and behavior. In European countries, for example, interacting with coworkers who experienced a divorce or a recent childbirth increases individuals’ likelihood of exhibiting the same behavior (Aberg 2009; Buyukkececi et al. 2020). Using the case of Japan, Yu and Kuo (2017) also argue that workplaces are primary venues for singles to be in contact with older and married people, who are likely to hold more traditional values about marriage than their friends. In the same way that being exposed to nontraditional family values raises individuals’ support for nontraditional family behavior (Choe et al. 2014; Rindfuss et al. 2004), being exposed to more traditional values may lead singles to want to marry more and seek partners more actively. Because jobs that require more interactions among coworkers are likely to expose singles to their older and married coworkers’ values more, such jobs may also strengthen their intention to marry. Using cross-sectional data, Yu and Kuo (2017) indeed find more sociable jobs to be associated with a greater desire for marriage among Japanese women, but a longitudinal analysis is necessary to rule out the possibility that those selecting into more sociable jobs viewed work and family differently to begin with. In the analysis, we specifically test how changes in one’s workplace’s emphasis on collaboration and teamwork correspond to shifts in one’s marital intention and partner-seeking behavior over time.

To summarize, prior research has offered several reasons for the trend toward later and fewer marriages. One explanation emphasizes the persistence of gender inequality and rising opportunity costs of marriage, leading to our expectation that Japanese women with promising job characteristics, such as high pay and abundant autonomy, will express a lower intention to marry and put less effort into seeking romantic partners. A second perspective focuses on how economic anxiety dampens singles’ interest in marriage. From this framework we derive the hypothesis that those with precarious job conditions, such as lacking job security and being in workplaces with frequent staff shortages and deadline pressure, will desire marriage and seek partners less. Because the Japanese context puts the financial burden associated with marriage and family almost entirely on men, we anticipate that precarious employment conditions will lower men’s intention to marry considerably more than women’s. Extending the argument about precarious job conditions we further propose that economic anxiety resulted from poor-quality jobs will prevent singles – especially men – who want to marry from taking actions to meet partners. We simultaneously consider the alternative possibility that inferior job conditions are especially likely to discourage those ambivalent about marriage from participating in activities that enable them to meet romantic partners. Finally, the diffusion of norms and values through social contacts is also thought to affect marital intentions. Based on the argument about the importance of social influences, we hypothesize that Japanese singles in workplaces that facilitate frequent interactions with older and married coworkers will develop a stronger intention to marry and take more actions to find partners.

## 3. Methods

### 3.1 Data

The study uses data from the Japan Life Course Panel Survey (JLPS), a longitudinal survey conducted annually since 2007 by the Institute of Social Science at the University of Tokyo. The JLPS includes young and middle-age cohorts, defined as those who were 20–34 and 35–40 years old in the initial wave, respectively. By design, the two cohorts can be combined to make up a larger sample (Yu and Kuo 2016, 2017). We pool the data from Waves 1–9 for both cohorts to create a person-year sample. The JLPS added a replenishment sample at Wave 5 to compensate for the modest attrition over time.^3^ Because incorporating data from a replenishment sample reduces the attrition-related bias of longitudinal survey data (Deng et al. 2013), we also include the replenishment sample in the person-year data from Wave 5 onward.

From the pooled sample we select all person-years prior to respondents’ first marriage for the analysis. The JLPS asks the unmarried to report their marital intentions and the actions they had taken to seek romantic partners at every wave, making it possible to observe how singles’ intentions and behaviors change with their job conditions. We focus on the period before first marriage because the considerations for first marriage tend to differ from those for remarriage. To observe within-person changes, we exclude respondents who were observed just once in the survey. After these selections, and after eliminating a small percentage of observations that have invalid information for key variables, the analytic sample contains of 6,061 person-years from 1,136 men and 5,656 person-years from 1,044 women.

### 3.2 Variables and measurement

The analysis contains two parts. The dependent variable for the first part is the intention to marry. We measure this intention based on the question whether respondents: (1) absolutely want to marry, (2) would like to marry if possible, (3) would be fine to either marry or not marry, (4) do not want to marry, or (5) have not thought about marriage. We combine responses (5) with (3), as they both indicate ambivalence toward marriage,^4^ and create a linear measure of marital intention, ranging from 1 to 4, with 4 being the strongest.

The second part of the analysis focuses on the actions singles take to pursue romantic partners. We use the number of partner-seeking activities engaged as the dependent variable. The JLPS asks single respondents whether they have recently taken each of an extended list of partner-seeking actions (14 in total), including asking parents or relatives to introduce potential partners, taking part in arranged dates,^5^ using matchmaking services, asking friends to introduce potential partners, asking coworkers or supervisors to introduce potential partners, participating in enrichment lessons and hobby meetings to meet the other sex, and trying to meet potential partners through the internet. We count the number of activities reported, considering engagement in more partner-seeking activities as being more active.

Of course, not all partner-seeking actions have the same intensity – using matchmaking agencies, for example, is a more direct and potentially faster way of finding a marriage partner than attending school club activities or taking enrichment lessons. Following prior research that differentiate partner-seeking activities into formal and informal ones, with the latter being less direct and imposing fewer obligations (Yu and Kuo 2016, 2017), we further analyze how job conditions may be associated with the uses of formal and informal search methods separately. Formal partner-seeking methods include taking part in arranged dates, attending matchmaking parties, asking parents or relatives to introduce potential partners, and using matchmaking agencies, all of which require individuals to be unequivocal about their intention to marry and be committed to trying out potential partners offered to them. We consider the other methods respondents reported to use to find partners as informal ones and use the number of informal methods reported as the dependent variable for the models about informal partner seeking. We use a binary indicator of whether respondents have been engaged in any formal search activities, instead of the count of activities, to analyze the use of formal partner-seeking methods. The reason we do so is that formal partner-search activities tend to require more commitment, making individuals less likely to be engaged in more than one at the same time. The small number of person-years during which respondents reported participating in more than one formal activities (2.1%) also makes it difficult to empirically compare these observations with those for which only one formal activity was reported.

For the main predictors, we include a series of attributes of respondents’ jobs. Although Japanese workers are known to experience relatively few employer changes in their careers (Yu 2010), it is common for them to change jobs within firms (Cheng and Kalleberg 1996). Within-firm job changes, along with recent increases in firm turnover rates and potential alterations in firm environments over time (Kambayashi and Kato 2017), lead to considerable shifts in each individual’s job conditions during the observed period.^6^ We can therefore rely on within-person variations to estimate the relationships between job characteristics and partnering intentions and behaviors. The first job attribute we introduce is earnings, which we approximate using respondents’ reports on their own income during the past year. Although respondents’ income may not be identical to their wages, research indicates that self-reported income is a close proxy for earnings in Japan (e.g., Yu 2012). The JLPS recorded income in 13 categories. We use the midpoint of each category to create a continuous measure, in the unit of one million yen (about US$9,200). Around 5% of the observations in the sample have no valid information for income. We imputed missing income with the most recent income record of respondents within the past three years, following previous research using longitudinal income data (Cheng 2014).^7^ Given the closeness of job earnings and personal income in Japan, we also use income and earnings somewhat interchangeably in our discussion of the results.

Next, we include job insecurity, the job’s potential for skill growth, and job autonomy. Job insecurity is derived from the question of how likely – (1) very much, (2) to some extent, (3) not so much, or (4) not at all – respondents will lose their jobs in the next year. We code the responses from 1 to 4, with 4 being the most insecure. For the job’s potential for skill growth, we create an index from averaging respondents’ assessments, reported on a 1–4 scale, of their opportunities to learn and their likelihood to accumulate skills at the job. We measure job autonomy with three items, also reported on a 1–4 scale, concerning the extent to which respondents can decide their own pace at work; the extent to which they can decide how to perform their jobs; and the extent to which they can adjust work schedules according to personal needs. We use the average response to create an index.

We also introduce the prevalence of teamwork, frequent staff shortages, and pressure from deadlines, measured with questions about respondents’ workplaces. Specifically, respondents were asked whether they agree or not that most work in their workplace is done collaboratively or by teams. We code the prevalence of teamwork as 1, otherwise as 0, if the answer is positive. The characteristic of frequent staff shortages is also coded as 1 or 0, based on the answers to whether respondents’ workplaces are constantly short of staff. Similarly, deadline pressure is coded as 1 or 0 according to whether respondents agree that their workplace always imposes stressful deadlines. For the final attribute, we use reports of daily working hours to measure time demands of respondents’ jobs.

Although research suggests that nonstandard employment may lower Japanese singles’ intention to marry (Piotrowski, Kalleberg, and Rindfuss 2015), we do not further differentiate workers by standard or nonstandard status. Our reason is that the models already account for the job attributes thought to make nonstandard employment affect marriage timing, such as earnings, security, working hours, and workplace staff shortages. Besides, the main results were similar when we accounted for nonstandard employment status in an exploratory analysis. Because the sample includes person-years when respondents were jobless, we also include a binary indicator of having a job at the observed time. To avoid multicollinearity, we center all the job characteristics at the sample median and code the jobless as 0 for these variables.^8^ With this transformation, the dummy for having a job is ultimately estimating the difference in the outcome between those with “average” jobs – i.e., job conditions equivalent to the median values – and those without jobs. Meanwhile, the coefficients for job attributes indicate how each unit of increase in a given job characteristic from the median contributes to the outcome of interest.^9^

The models contain several time-varying controls, such as the level of education completed (high school and less, junior or vocational college, four-year university and above), current enrollment in school,^10^ and whether respondents were romantically involved. To account for the different norms and partnering opportunities in areas with different levels of urbanization, we also control for whether respondents live in: (1) major population centers, (2) large cities (but not major population centers), (3) other cities, or (4) towns or villages at the survey time.

For some models, we also include respondents’ self-identified chances to meet potential dating partners. One proposition we test is that jobs requiring frequent interactions with coworkers will expose singles to traditional family values more, thereby raising the interest in marriage. Introducing the opportunities to meet potential partners enables us to show whether the prevalence of teamwork contributes to the intention to marry by enhancing singles’ opportunities to meet other singles through work, rather than exposing singles to traditional family values. The JLPS asked whether respondents: (1) hardly, (2) not so frequently, (3) somewhat frequently, or (4) frequently had chances to meet people whom they are interested in dating. We code the responses from 1 to 4 based on these categories. Because this question was not asked in Wave 2, the models including the opportunities to meet potential partners must rely on a more restricted sample. To provide more information, Table 1 presents descriptive statistics for all variables used in the study.

### 3.3 Analytic strategy

The analysis uses fixed-effects models, expressed as follows:

(1) $${\text{intention}}_{it}=\gamma_{0}+\Sigma a_{j}JOB_{jit}+\Sigma b_{k}X_{kit}+u_{i}+{\text{year}}_{t}+\varepsilon_{it},$$

where the outcome is the expressed intention to marry of person *i* at time *t;* γ_0_ is the intercept; *JOB*_*jit*_ represents *j* job characteristics (working hours, job insecurity, job autonomy, etc.); Σα_*j*_ denotes the coefficients of job characteristics; *X*_*kit*_ is a vector of time-varying control variables (e.g., education, school enrollment, wave dummies); Σ*b*_*j*_ denotes the coefficients of this vector of variables; *u*_*i*_ represents individual fixed effects while *year*_*t*_ survey-year fixed effects; and ε_*it*_ indicates the error term.^11^ The inclusion of individual fixed effects allows the models to account for all time-invariant individual characteristics, such as general personality traits and overall preferences regarding work and family. Thus, unlike previous research with cross-sectional data (Yu and Kuo 2017), we can better address the possibility that unobserved personal attributes explain both job choices and marital intentions. With survey-year fixed effects, the models further adjust for year-to-year differences that may affect considerations about marriage, such as macroeconomic fluctuations.^12^

We use the same models for the second part of the analysis, except for changing the outcome to the number of partner-seeking activities engaged. We also add the intention to marry at the right-hand side to investigate whether job attributes are associated with partner-search behavior even after accounting for this intention. To show whether job quality may weaken the link between the intention to marry and partner seeking, we further interact the intention with job attributes in the models. We use similar models to examine the number of informal partner-seeking activities participated and the use of formal partner-search methods. Because the latter outcome is dichotomous, we also fit random-effects logit models to ensure that the results are not sensitive to our choice of linear regression models. We opt not to use fixed-effects logit models because unlike linear fixed-effects models, they require elimination of all individuals whose outcomes do not change across time, making the sample too small to yield stable results.^13^

## 4. Results

Table 2 shows results from fixed-effects models predicting single men’s and women’s marital intentions. Results in Models 1 and 2 for both groups are similar, indicating that romantic involvement hardly mediates the associations between job attributes and marital intention status. Among Japanese men, having an “average” job (i.e., each job trait equal to the sample median) is associated with a stronger intention to marry than having no job. Having a higher income increases men’s intention to marry, whereas having a relatively insecure job and being in a workplace that is constantly short of staff decrease this intention. These findings provide support for the argument that lacking economic stability dampens men’s marriage desire.

Table 2 also indicates that greater job autonomy increases men’s intention to marry. This finding is interesting because job autonomy, which helps ease work-family conflict, is typically thought to raise women’s, not men’s, interest in marriage (Kuo and Raley 2016b). As married men rarely share domestic work in Japan, the relationship between job autonomy and marital intention status for men is unlikely related to the expectation of work-family conflict. Perhaps in typically group-oriented Japanese workplaces, the ability to arrange tasks and schedules conveys a sense of privilege. Men with greater job autonomy may therefore feel more positive about their job prospects, leading to a stronger marital intention.

Precarious working conditions, such as having a job that may terminate within a year and working in firms that face frequent staff shortages, do not affect women’s marital intentions as they do men’s (p < 0.05 for the gender differences). Although a higher income seems to be associated with a stronger intention to marry in Model 1, the association weakens after including the partnering status (Model 2). Overall, the job characteristics relevant to economic prospects play almost no role in how much women desire marriage. Thus, to the extent that Japanese women assess the opportunity costs of marriage based on their current job conditions, their intention to marry is rather independent of these costs.

At the same time, being in a workplace where teamwork is prevalent enhances women’s desire for marriage. This result is consistent with the argument that norms and values spread through social encounters play a role in shaping marital intentions. We argue that in workplaces where most work is done collaboratively, singles likely have more opportunities to interact with married or older workers, who lead them to adopt more traditional views on marriage. When we add respondents’ reported chances to meet potential partners in Model 3 (which leads to a reduction of sample size because this item was omitted at Wave 2), women’s marital intention continues to be stronger in workplaces where teamwork is prevalent. Thus, the association between teamwork prevalence and the intention to marry cannot be explained by singles’ potentially more opportunities to meet people whom they want to date, making the value-based interpretation more likely. In contrast to women, being in a collaborative or teamwork-oriented workplace is hardly related to men’s marital intention status.

Aside from job attributes, two other findings in Table 2 are notable. First, having a steady partner is strongly associated with the intention to marry for both men and women. It is possible that a rising desire for marriage makes singles put more effort into obtaining and maintaining romantic relationships. Nevertheless, the second notable finding, that having more chances to meet potential partners is linked to a stronger marital intention, suggests that it is having a relationship that increases the desire for marriage. Both being romantically involved and meeting many potential partners enhance the feasibility of marriage. The fact that both variables are positively tied to the intention to marry suggests that Japanese singles’ intention to a considerable extent depends on how feasible they believe marriage is for them.

Turning to partner-seeking behavior, Table 3 presents fixed-effects models predicting the number of activities Japanese men have been engaged for the sake of meeting potential partners. We begin with a baseline model and then add the intention to marry. Model 3 further examines how job conditions moderate the link between marital intention status and partner-seeking behavior. In an earlier analysis, we included interactions between marital intention status and all job characteristics. For simplicity, we only keep the interactions for which the p-value is smaller than 0.10 in the model presented (Model 3).

According to Model 2 in Table 3, income is positively linked to the number of partner-seeking activities engaged. A stronger marital intention is also positively associated with the number of partner-seeking activities. Model 3, however, indicates that this association depends on income. Likewise, the relationship between the intention to marry and partner-seeking activities is moderated by whether men frequently face deadline pressure in the workplace. To illustrate these results more intuitively, Figure 2 presents the predicted changes in the number of partner-seeking activities engaged with marital intention status for those with low and high income levels (i.e., income at the 10th and 90th percentile) and for those facing frequent deadline pressure and not. We hold all other variables at the sample mean to calculate the predicted values. The figure indicates that having a high income and facing low deadline pressure especially increase the number of partner-search activities participated by those with little interest in marriage. In other words, the patterns in Figure 2 are more similar to the one presented in Panel B in Figure 1. Men eager to marry would take actions to find partners regardless of their income or workplace deadline pressure. It is those ambivalent about marriage who would disproportionately withdraw themselves from seeking romantic partners when having low-paying and stressful jobs.

Table 4 presents results from fixed-effects models predicting men’s involvement in informal partner search. Model 1 indicates that a stronger intention to marry is positively associated with the number of informal search activities engaged, but job conditions are hardly relevant. In Model 2, we add all the interactions between marital intention status with job characteristics that have p-values smaller than 0.10. The model demonstrates that the link between marital intention status and the number of informal search activities depends on men’s income, experience of deadline pressure in the workplace, and work hours. The results about income and workplace deadline pressure are similar to those shown in Model 3 in Table 3, indicating that changes in Japanese men’s informal partner-search activities according to their income and deadline pressure levels largely drive the patterns in Figure 2. Thus, similar to what Figure 2 shows, men most eager to marry are likely to adopt multiple informal methods to seek partners regardless of their income and whether their workplace imposes frequent deadlines. Men who express little interest in marriage, however, would be engaged in especially few informal search activities when they receive low income or face considerable deadline pressure.

**Figure 3:**
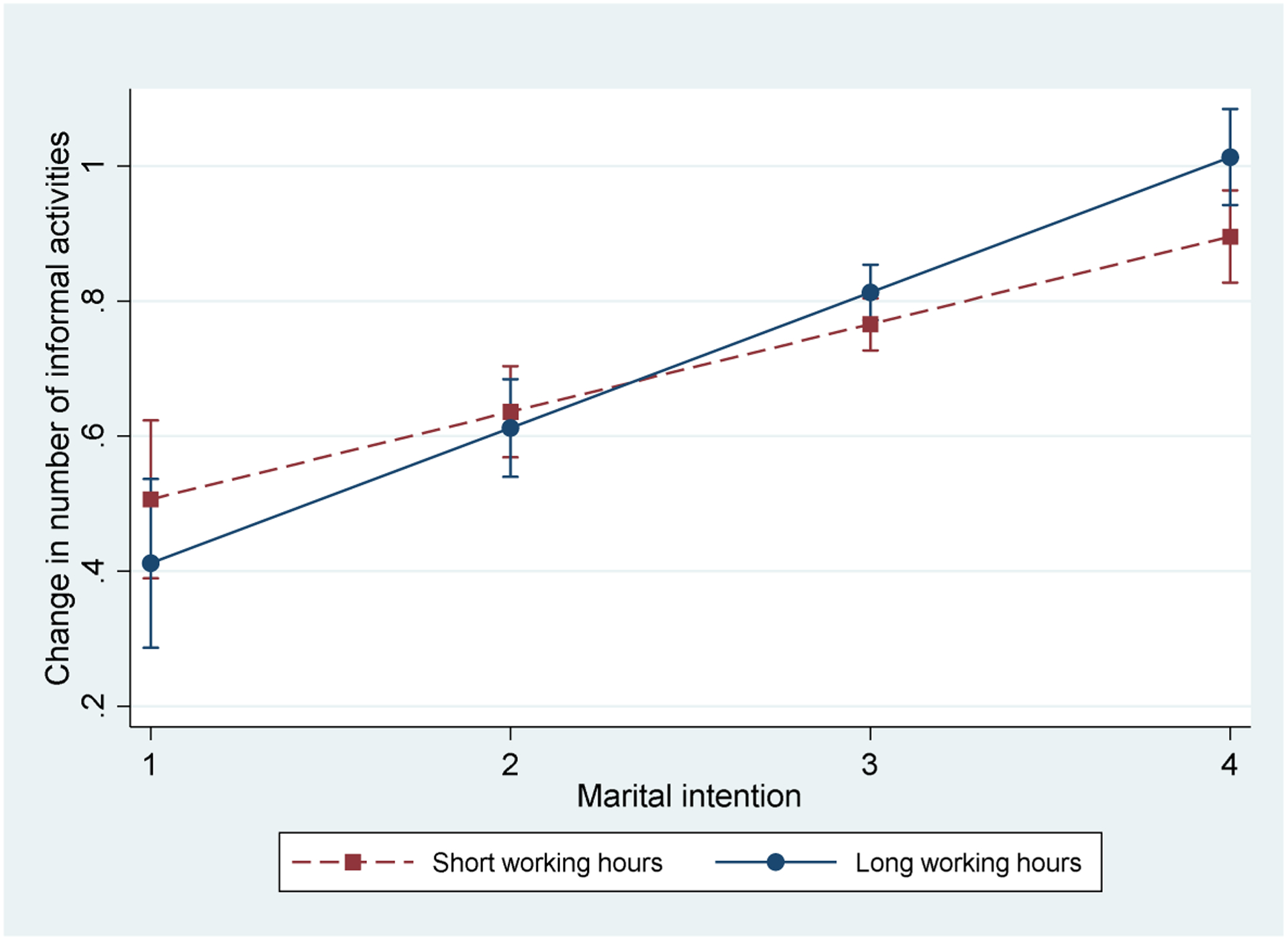
Predicted numbers of informal partner-search activities by work hours and marital intention for men *Note*: The predicted probabilities are calculated using coefficients in Model 2 for informal partner search in Table 4. The predicted values are presented with 95% confidence intervals. Because overlapped confidence intervals do not indicate no difference between the predicted values, we also test the difference between each pair of predicted values is statistically different from zero. The p-value for the test is smaller than 0.05 for the pair at the highest level of marital intention (marital intention = 4).

Model 2 in Table 4 also indicates that work hours moderate how men with different intention statuses participate in informal search activities. Figure 3 illustrates changes in men’s number of informal search activities according to their marital intention statuses and work hours, with all other variables held at the sample mean. We use the values at the 90^th^ and 10^th^ percentile to represent long and short work hours, respectively. The figure shows that working long hours does not weaken the tendency for men who desire marriage more to participate in more informal search activities. In fact, men with a strong intention to marry (marital intention status = 4) would be engaged in more informal search activities when they work extended hours (11 hours per day) than relatively short hours (7 hours per day).^14^ This finding seems counterintuitive because those working long hours should have less time for informal partner-seeking activities. One possible explanation is that men working excessive hours more likely feel the need to take deliberate actions to meet potential partners if they want to marry, given that their long working hours may deprive them from having regular social activities in which they could meet other singles.

**Figure 4:**
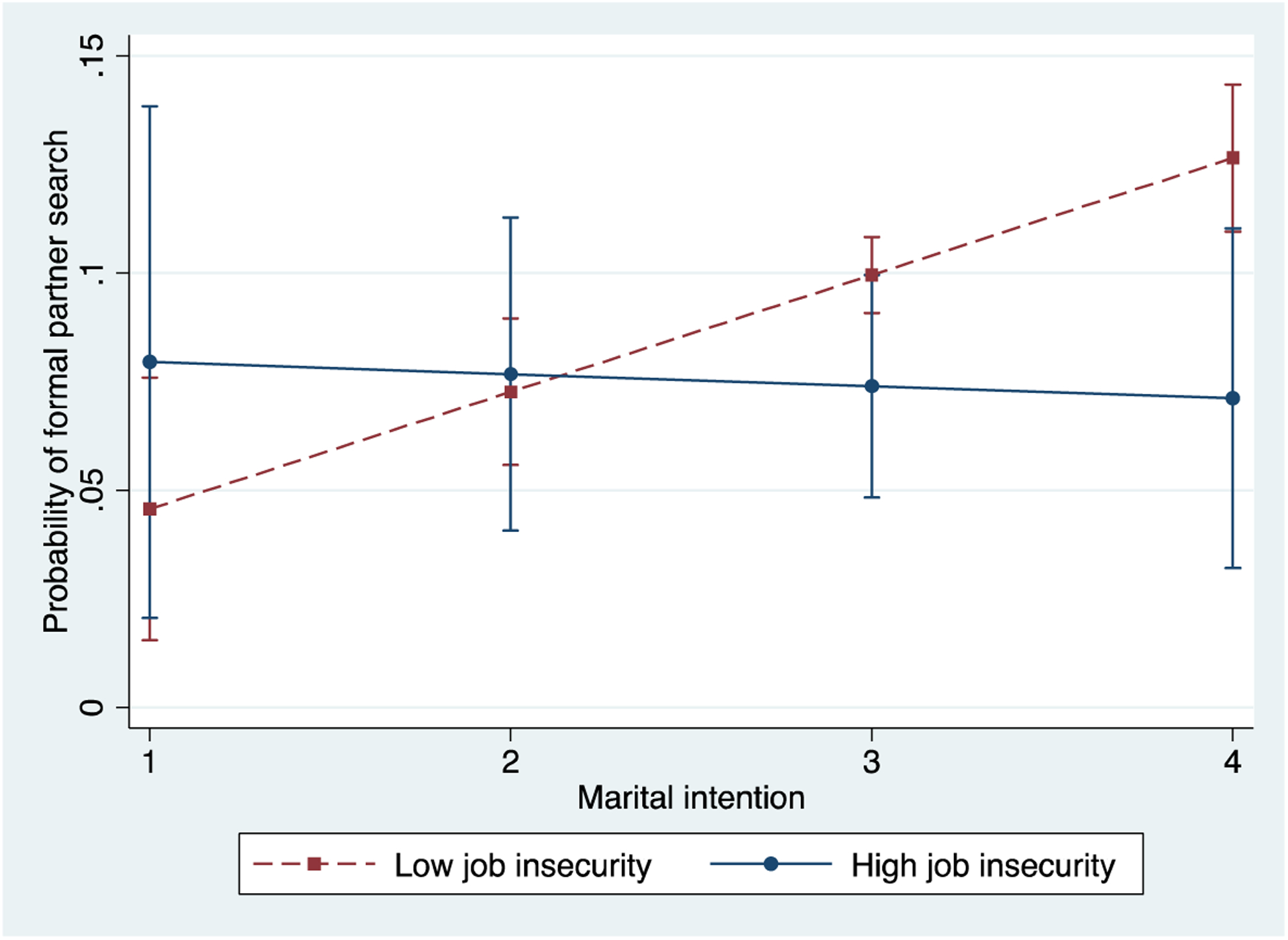
Predicted probabilities of formal partner search for men of varying levels of job security and marital intention *Note*: The predicted probabilities are calculated using coefficients in Model 2 for formal partner search in Table 4. The predicted values are presented with 95% confidence intervals. Because overlapped confidence intervals do not indicate no difference between the predicted values, we also test the difference between each pair of predicted values is statistically different from zero and found the p-value smaller than 0.05 for the pair at the highest level of marital intention (i.e., marital intention = 4).

Table 5 presents results from fixed-effects models predicting men’s use of formal partner-search methods, such as relying on matchmaking agencies, taking part in arranged dates, and asking parents or relatives to introduce potential partners. We also include results from random-effects logit models, which demonstrate similar patterns to those from the fixed-effects models. Specifically, we find that men with a higher income are much more likely to be engaged in any formal partner-search activities (Models 1 and 3). Although job insecurity is not associated with formal partner search universally, the interaction term in Model 2 (as well as in Model 4) suggests that it is somewhat connected to how likely men will act on their intention to marry. Like for previous figures, we use coefficients from Model 2 to calculate the predicted probabilities for men with high job insecurity (very much likely to lose their job) and low job insecurity (not at all likely to lose their job), with all variables other than marital intention status held at the sample mean. Figure 4 shows these probabilities. Interestingly, the pattern here is somewhat different from those in the previous figures. Having high job insecurity appears to especially lower the probability that men eager to marry will use a formal method to find a marriage partner (p < 0.05 when comparing the difference with zero for men with the strongest marital intention). Together with the result that a higher income increases the probability of taking part in a formal partner-seeking activity, this finding suggests that a lack of economic stability lowers single men’s likelihood to use formal partner-seeking methods in particular, perhaps because there is far more scrutiny of men’s financial status in formal matchmaking activities (Yu and Hertog 2018). Thus, even men eager to marry would avoid this route when they fail to meet certain economic expectations.

Turning to women, Table 6 shows models predicting women’s number partner-seeking activities engaged and their involvement in informal and formal partner search. Similar to men, women with a stronger intention to marry are engaged in more partner-seeking activities. Although having an average job, as opposed to no job, increases the number of partner-seeking activities in which women take part, most job attributes are barely relevant. When we separate informal from formal partner-search methods, we nevertheless find that being in a workplace where teamwork is prevalent is positively associated with women’s probability of using a formal method to meet a partner. This finding is robust regardless of whether we use random-effects logit or fixed-effects linear regressions. Earlier we showed that the prevalence of teamwork in the workplace is positively linked to women’s desire for marriage. The result here indicates that a shift to a job that requires much teamwork also increases women’s use of formal partner-search methods, which are generally considered as more traditional ways of finding partners. Because more conventional family values could also facilitate more traditional partner-seeking behavior, this finding provides additional support that frequent contacts with coworkers are likely to expose single women to conventional family values more.

In models not shown here, we also tested whether the associations between marital intention status and partner-seeking behavior for women depend on their job attributes (i.e., adding the interactions between marital intention status and job attributes to the models). We found the coefficients for the interactions negligible, indicating that job attributes do not moderate the relationships between women’s intention to marry and their partner-seeking behavior.

## 5. Conclusions

Prior researchers explaining later and fewer marriages in Japan have suggested a few narratives, each of which has different implications for how alterations in job attributes may lead to changes in singles’ intention to marry and actions taken to seek marriage partners. Results from our study provide support for the narrative that focuses on economic stagnation and deterioration of job quality for men, as men facing more precarious working conditions, including low pay, high insecurity, limited autonomy, and a constant staff shortage in the workplace, express lower levels of desire for marriage. Despite some prior research showing that having unstable jobs and low income are increasingly likely to deter Japanese women’ transition to first marriage (Fukuda 2013; Piotrowski, Kalleberg, and Rindfuss 2015), we find little evidence that precarious job conditions dampen women’s intention to marry. One possible explanation is that our use of fixed-effects models helps take into account unobserved personal traits that could lead to both high-paying stable jobs and a strong marital intention, such as having the drive to achieve socially approved status at every life-course stage. Once such traits are accounted for, job prospects hardly influence women’s interest in marriage. Alternatively, it is also possible the previous findings of the faster paces of marriage transitions for women with higher-paying and more stable jobs reflect how women of different economic circumstances are received in the marriage market, not how they themselves consider marriage. Regardless, our findings suggest that Japanese women do not view their achieving a certain level of economic stability as a precondition for marriage. Our results are also inconsistent with the account that Japanese women intentionally delay or reject marriage because of the opportunity costs of marriage, as women have more to lose with marriage are no different in their desire to marry.

Rather than having a promising or stable job, we find that having a job that facilitates frequent contacts with coworkers is conducive to a stronger intention to marry among Japanese women. We argue that this finding is consistent with the account that emphasizes norms and values and how social contacts help spread values. Although we do not have specific information on the composition of workers in respondents’ workplaces, Japanese firms often consist of workers of a wide age range and encourage interactions between the young and old (Yu and Kuo 2017). Thus, in workplaces where most work is done collaboratively, single women are more likely exposed to their older and married coworkers’ potentially more conventional family values. That being in such workplaces also increases single women’s likelihood to use a formal, more traditional method to seek marriage partners is congruent with the argument that the more frequent contacts with people who likely hold traditional family views make women more conventional about marriage and partner search.

Based on our results on job characteristics and the intention to marry, we can infer that economic conditions are imperative to men’s desire for marriage in Japan, whereas social influences play a critical role in shaping women’s. Although Yu and Kuo (2017) have suggested a similar gender divide, the present study is the first to provide longitudinal evidence to substantiate this claim. Because fixed-effects models rely exclusively on intra-person variation over time, we can be certain that men’s desire for marriage indeed rises with shifts in job conditions that raise their economic prospects, while women become more eager to marry when they switch to workplaces that emphasize teamwork. Therefore, we can conclude that the associations between precarious job conditions and marital intentions for men and the association between workplace social influences and marital intentions for women are not merely spurious.

Beyond providing longitudinal evidence, this study also makes a novel contribution by examining how job attributes moderate the relationships between marital intention status and actions taken to find a romantic partner. The analysis indicates that for Japanese men, having suboptimal job conditions, such as low pay and great pressure from deadlines, disproportionately discourages those less eager to marry from trying to meet romantic partners; those with a strong intention to marry would take actions even if their job conditions are not ideal. Thus, we need to pay attention to both marital intentions and job quality to understand Japanese men’s partner-seeking behavior.

As we show that some undesirable job traits discourage partner seeking especially for men with a low desire for marriage, we should note that precarious job attributes are also linked to men’s lower interest in marriage. Taken together, results from this analysis inform us the multiple ways in which the rising inequality in work conditions in Japan hampers marriage chances for men who are relatively disadvantaged in the labor market. Aside from being seen as unmarriageable by potential mates, such men are likely to develop a lower desire to marry. This lack of desire, in conjunction with their undesirable job traits, further lead these men to drastically withdraw from activities that could facilitate their meeting romantic partners, making marriage formation even more difficult.

By revealing that precarious job conditions can harm Japanese men’s marriage chances beyond decreasing their popularity among potential partners, this study enriches our understanding of the demographic implications of gender specialization within marriage. It is precisely because Japan’s marriage contract requires men to shoulder financial responsibilities nearly entirely, men with precarious job conditions tend to internalize their inability to provide and, in turn, retreat from marriage. With the continuation of the rigid gender division of labor within marriage and rising deterioration of job quality in Japan, we may find a growing number of men who give up on the idea of marriage. Replacing marriage with cohabitation, as observed among low-income men and women in the United States (Edin and Kefalas 2005), is nevertheless unlikely in Japan, because cohabitation, as a precursor to marriage (Raymo et al. 2015), is likely to bring similar gender expectations as marriage for heterosexual couples. If this is the case, we should also expect Japan’s fertility rate to decline further in the future.

Beyond Japan, this research has general implications for other East Asian societies that have undergone similar marriage trends. For example, our argument that women’s considerations about marriage are socially determined, whereas men’s are economically driven may be applicable in other societies that share certain cultural legacies with Japan. If this argument holds elsewhere, it would help explain why the trend toward later and fewer marriages are observed across many East Asian countries in spite of their varying degrees of gender inequality, which make the economic costs of marriage differ considerably for women among those countries (Raymo et al. 2015; Yu 2009). In order to know how prevalent this gender divide is across societies, future research on marriage trends needs to more explicitly focus on men’s and women’s desires for marriage, not just their timing of marriage, and to more often consider how economic and noneconomic factors may separately shape men’s and women’s marital intentions.

Finally, this study underscores the importance of job conditions to our understanding of marriage considerations and timing. Although much previous research on marriage formation has examined the roles of earnings (e.g., Fukuda 2013; Oppenheimer, Kalmijn, and Lim 1997; Sweeney 2002), our findings indicates that other job traits, such as the workplace’s staffing adequacy, the job’s deadline pressure, and the prevalence of teamwork in the workplace, can similarly shape marital intentions and partner-seeking actions. Future researchers aiming to explain how work affects marriage and family formation should put extra effort into collecting and examining detailed characteristics of individuals’ jobs.

## Figures and Tables

**Table 1:** Descriptive statistics of the analytic sample

	Total	Men	Women
Marital intention (1–4)	3.0 (.9)	3.0 (.9)	3.1 (.9)
Number of partner-seeking activities engaged	.9 (1.3)	.9 (1.3)	.9 (1.3)
Number of informal partner-seeking activities engaged	.8 (1.1)	.8 (1.2)	.7 (1.1)
Involved in formal partner-seeking activities (%)	10.7	9.4	12.0
Work status (%):			
With a job	89.9	88.9	90.9
Without a job	10.2	11.1	9.1
Income (1,000,000 yen)	2.8 (2.0)	3.0 (2.2)	2.5 (1.6)
Job insecurity (1–4)^a^	1.6 (.8)	1.7 (.8)	1.6 (.8)
Job enabling skill growth (1–4)^a^	2.5 (.9)	2.5 (.8)	2.4 (.9)
Job autonomy (1–4)^a^	2.4 (.7)	2.5 (.7)	2.4 (.7)
Teamwork prevalent in workplace (0–1)^a^	.5 (.5)	.5 (.5)	.5 (.5)
Workplace staff shortage (0–1)^a^	.3 (.5)	.3 (.5)	.3 (.5)
Deadline pressure (0–1)^a^	.2 (.4)	.2 (.4)	.2 (.4)
Daily work hours^a^	8.7 (2.1)	9.0 (2.2)	8.4 (1.9)
Education (%):			
High school and less	29.3	33.9	24.3
Junior/vocational college	30.7	20.6	41.5
University and more	40.1	45.5	34.2
Enrolled in school (%):	7.5	8.1	6.8
Residential location (%):			
Major population center	39.5	42.9	35.8
Large city	22.2	19.3	25.3
Other city	30.9	31.1	30.7
Town/village	7.4	6.7	8.2
Chance to meet potential partners (1–4)	1.7 (.7)	1.7 (.7)	1.7 (.6)

a Based on the original scores reported by all those with a job.

*Note*: The unit of analysis is person-years. The sample contains 6,061 person-years for men and 5,656 person-years for women. The numbers of observations are smaller for “chance to meet potential partners” because the item was not included in Wave 2. All numbers followed by parentheses are mean values, with the standard deviation in the parenthesis; other values are in percentage (as indicated in the labels).

**Table 2:** Results from fixed-effects models predicting marital intention status

	Men					
	Model 1		Model 2		Model 3	
	Coef. (SE)	*p*	Coef. (SE)	*p*	Coef. (SE)	*p*
Having a job	.089 (.037)	.016	.086 (.037)	.019	.079 (.041)	.052
Income	.017 (.007)	.011	.016 (.007)	.016	.021 (.008)	.006
Job insecurity^a^	−.031 (.012)	.008	−.029 (.012)	.014	−.042 (.013)	.001
Job enabling skill growth^a^	.010 (.013)	.438	.008 (.013)	.538	.009 (.015)	.543
Job autonomy^a^	.064 (.015)	.000	.063 (.015)	.000	.068 (.017)	.000
Teamwork prevalent in workplace^a^	.012 (.019)	.514	.012 (.019)	.512	.017 (.021)	.421
Workplace staff shortage^a^	−.046 (.020)	.023	−.049 (.020)	.016	−.058 (.022)	.010
Deadline pressure^a^	.004 (.024)	.879	.002 (.024)	.947	−.014 (.026)	.594
Daily work hours^a^	.004 (.005)	.430	.004 (.005)	.394	.010 (.006)	.098
Having a steady partner			.141 (.025)	.000	.164 (.028)	.000
Chance to meet potential partners					.062 (.017)	.000
Education (*ref.* High school and less):						
Junior/vocational college	.071 (.066)	.276	.065 (.065)	.317	.044 (.085)	.608
University and above	−.051 (.063)	.419	−.055 (.063)	.383	−.042 (.073)	.567
Enrolled in school	.017 (.044)	.693	.013 (.044)	.769	−.014 (.051)	.781
Large city	.146 (.059)	.014	.144 (.059)	.015	.136 (.065)	.035
Other city	.042 (.059)	.475	.042 (.059)	.482	.032 (.064)	.622
Town/village	.118 (.090)	.188	.117 (.090)	.192	.093 (.099)	.350
Constant	2.864 (.059)	.000	2.843 (.059)	.000	2.733 (.072)	.000
N of person-years	6,061		6,061		5,144	
N of respondents	1,136		1,136		1,121	

	Women					
	Model 1		Model 2		Model 3	
	Coef. (SE)	*p*	Coef. (SE)	*p*	Coef. (SE)	*p*
Having a job	.005 (.037)	.883	.012 (.037)	.754	−.012 (.041)	.772
Income	.013 (.009)	.149	.012 (.009)	.201	.021 (.010)	.034
Job insecurity^a^	.001 (.011)	.901	.001 (.011)	.897	−.004 (.012)	.757
Job enabling skill growth^a^	.003 (.012)	.803	.005 (.012)	.697	.010 (.014)	.465
Job autonomy^a^	.024 (.015)	.108	.023 (.015)	.123	.021 (.016)	.194
Teamwork prevalent in workplace^a^	.040 (.018)	.030	.039 (.018)	.034	.047 (.020)	.021
Workplace staff shortage^a^	.027 (.020)	.175	.022 (.020)	.267	.020 (.022)	.349
Deadline pressure^a^	−.002 (.025)	.948	−.001 (.025)	.956	.014 (.027)	.597
Daily work hours^a^	−.001 (.006)	.933	−.001 (.006)	.909	−.001 (.007)	.837
Having a steady partner			.107 (.021)	.000	.104 (.023)	.000
Chance to meet potential partners					.044 (.018)	.013
Education (*ref.* High school and less):						
Junior/vocational college	−.040 (.064)	.540	−.044 (.064)	.494	−.025 (.081)	.763
University and above	.150 (.064)	.019	.146 (.064)	.022	.087 (.072)	.228
Enrolled in school	.083 (.042)	.051	.082 (.042)	.054	.067 (.049)	.173
Residential location (*ref.* Major population center):						
Large city	.076 (.057)	.181	.072 (.057)	.206	.125 (.061)	.039
Other city	−.129 (.059)	.027	−.130 (.058)	.025	−.108 (.064)	.092
Town/village	−.113 (.091)	.218	−.121 (.091)	.184	−.097 (.100)	.334
Constant	3.006 (.062)	.000	2.980 (.062)	.000	2.870 (.075)	.000
N of person-years	5,656		5,656		4,831	
N of respondents	1,044		1,044		1,035	

a Centered at the sample median and coded as zero for those without jobs.

*Note*: Values in parentheses are standard errors. The models also include dummies for each survey round, but the coefficients are omitted to conserve space. Following *Demographic Research*’s guidelines, we present p-values instead of symbols referring to discretized p-value intervals.

**Table 3:** Fixed-effects models predicting the number of partner-seeking activities engaged by men

	Model 1		Model 2		Model 3	
	Coef. (SE)	*p*	Coef. (SE)	*p*	Coef. (SE)	*p*
Marital intention			.195 (.027)	.000	.235 (.040)	.000
Having a job	.067 (.071)	.339	.051 (.070)	.471	.050 (.070)	.473
Income	.045 (.013)	.001	.041 (.013)	.001	.118 (.035)	.001
Income × marital intention					−.024 (.010)	.019
Job insecurity^a^	−.021 (.022)	.347	−.015 (.022)	.488	−.016 (.022)	.477
Job enabling skill growth^a^	−.007 (.026)	.778	−.009 (.025)	.730	−.009 (.025)	.735
Job autonomy^a^	.032 (.029)	.270	.020 (.029)	.494	.020 (.029)	.479
Teamwork prevalent in workplace^a^	.044 (.036)	.232	.041 (.036)	.257	.044 (.036)	.229
Workplace staff shortage^a^	−.040 (.039)	.308	−.030 (.039)	.436	−.033 (.039)	.393
Deadline pressure^a^	.008 (.046)	.859	.008 (.045)	.863	−.400 (.146)	.006
Deadline pressure^a^ × marital intention					.135 (.046)	.003
Daily work hours^a^	.008 (.010)	.396	.008 (.010)	.444	.008 (.010)	.410
Having a steady partner	−.210 (.048)	.000	−.237 (.048)	.000	−.235 (.048)	.000
Education (*ref.* High school and less):						
Junior/vocational college	.100 (.126)	.426	.087 (.125)	.485	.081 (.125)	.517
Completed university or higher	−.035 (.121)	.770	−.025 (.120)	.838	−.017 (.120)	.887
Enrolled in school	.028 (.084)	.739	.025 (.083)	.760	.029 (.083)	.732
Residential location (*ref.* Major population)						
Large city	.190 (.114)	.094	.162 (.113)	.151	.154 (.113)	.172
Other city	.050 (.114)	.661	.042 (.113)	.712	.049 (.113)	.661
Town/village	−.086 (.172)	.616	−.109 (.171)	.525	−.111 (.171)	.518
Constant	1.431 (.114)	.000	.878 (.137)	.000	.749 (.163)	.000
N of person-years	6,061		6,061		6,061	
N of respondents	1,136		1,136		1,136	

a Centered at the sample median and coded as zero for those without jobs.

Note: Values in parentheses are standard errors. The models also include dummies for each survey round, but the coefficients are omitted to conserve space. Following *Demographic Research*’s guidelines, we present p-values instead of symbols referring to discretized p-value intervals.

**Table 4:** Fixed-effects models predicting the number of informal partner-search activities engaged by men

	Model 1		Model 2	
	Coef. (SE)	*p*	Coef. (SE)	*p*
Marital intention	.165 (.024)	.000	.207 (.035)	.000
Having a job	.047 (.061)	.437	.047 (.061)	.444
Income	.022 (.011)	.051	.103 (.031)	.001
Income × marital intention			−.026 (.009)	.005
Job insecurity^a^	−.009 (.019)	.644	−.008 (.019)	.693
Job enabling skill growth^a^	−.002 (.022)	.946	−.002 (.022)	.917
Job autonomy^a^	.026 (.025)	.292	.027 (.025)	.278
Teamwork prevalent in workplace^a^	.040 (.031)	.208	.043 (.031)	.171
Deadline pressure^a^	−.007 (.039)	.864	−.260 (.128)	.043
Deadline pressure^a^ × marital intention			.083 (.041)	.040
Workplace staff shortage^a^	−.019 (.034)	.573	−.020 (.034)	.559
Daily work hours^a^	.012 (.009)	.158	−.041 (.027)	.125
Work hours^a^ × marital intention			.018 (.008)	.035
Having a steady partner	−.217 (.042)	.000	−.215 (.042)	.000
Constant	.843 (.119)	.000	.707 (.142)	.000
N of person-years	6,061		6,061	
N of respondents	1,136		1,136	

a Centered at the sample median and coded as zero for those without jobs.

*Note*: Values in parentheses are standard errors. The models also include dummies for each survey round, educational level, whether respondents were in school, and residential location, but the coefficients are omitted to conserve space. Following *Demographic Research*’s guidelines, we present p-values instead of symbols referring to discretized p-value intervals.

**Table 5:** Fixed- and random-effects models predicting men’s use of a formal partner-search method

	Fixed-effects models				Random-effects logit models			
	Model 1		Model 2		Model 3		Model 4	
	Coef. (SE)	*p*	Coef. (SE)	*p*	Coef. (SE)	*p*	Coef. (SE)	*p*
Marital intention	.021 (.007)	.002	.027 (.007)	.000	.780 (.103)	.000	.893 (.122)	.000
Having a job	.002 (.017)	.893	.000 (.017)	.993	.269 (.337)	.424	.243 (.339)	.473
Income	.012 (.003)	.000	.012 (.003)	.000	.250 (.037)	.000	.252 (.038)	.000
Job insecurity^a^	−.008 (.005)	.131	.021 (.016)	.196	−.204 (.095)	.031	.413 (.348)	.236
Job insecurity^a^ × marital intention			−.010 (.005)	.059			−.192 (.105)	.068
Job enabling skill growth^a^	−.000 (.006)	.982	−.000 (.006)	.999	.051 (.099)	.607	.053 (.099)	.591
Job autonomy^a^	−.012 (.007)	.084	−.012 (.007)	.084	−.161 (.111)	.149	−.166 (.112)	.137
Teamwork prevalent in workplace^a^	.002 (.009)	.790	.002 (.009)	.776	.072 (.144)	.618	.074 (.145)	.610
Deadline pressure^a^	.010 (.011)	.376	.010 (.011)	.356	.181 (.172)	.292	.182 (.172)	.288
Workplace staff shortage^a^	−.010 (.009)	.271	−.010 (.009)	.271	−.087 (.156)	.580	−.086 (.156)	.581
Daily work hours^a^	−.003 (.002)	.200	−.003 (.002)	.173	−.054 (.040)	.174	−.054 (.040)	.173
Having a steady partner	−.012 (.012)	.289	−.012 (.012)	.292	−.761 (.187)	.000	−.763 (.188)	.000
Constant	.034 (.033)	.301	.017 (.034)	.626	−6.956 (.524)	.000	−7.312 (.568)	.000
N of person-years	6,061		6,061		6,061		6,061	
N of respondents	1,136		1,136		1,136		1,136	

a Centered at the sample median and coded as zero for those without jobs.

*Note*: Values in parentheses are standard errors. The models also include dummies for each survey round, educational level, whether respondents were in school, and residential location, but the coefficients are omitted to conserve space. Following *Demographic Research*’s guidelines, we present p-values instead of symbols referring to discretized p-value intervals.

**Table 6:** Models predicting women’s partner-seeking actions

	Number of activities engaged				Number of informal activities		Use of formal search methods			
	Model 1		Model 2				Fixed effects		Random-effects logit	
	Coef. (SE)	*p*	Coef. (SE)	*p*	Coef. (SE)	*p*	Coef. (SE)	*p*	Coef. (SE)	*p*
Marital intention			.161 (.030)	.000	.117 (.025)	.000	.025 (.009)	.003	.683 (.088)	.000
Having a job	.163 (.076)	.031	.162 (.075)	.032	.151 (.062)	.015	.006 (.021)	.771	−.195 (.257)	.449
Income	.027 (.019)	.143	.025 (.019)	.171	.021 (.015)	.177	.002 (.005)	.735	.080 (.050)	.108
Job insecurity^a^	.010 (.023)	.660	.010 (.023)	.666	.007 (.019)	.719	.004 (.006)	.532	.082 (.072)	.251
Job enabling skill growth^a^	−.005 (.025)	.839	−.006 (.025)	.814	−.005 (.021)	.810	−.010 (.007)	.172	−.085 (.077)	.270
Job autonomy^a^	.056 (.030)	.060	.053 (.030)	.078	.039 (.025)	.117	.002 (.008)	.848	−.027 (.094)	.773
Teamwork prevalent in workplace^a^	.049 (.038)	.194	.042 (.037)	.257	.023 (.031)	.461	.027 (.011)	.011	.315 (.124)	.011
Workplace staff shortage^a^	.028 (.040)	.494	.024 (.040)	.550	.014 (.033)	.679	.008 (.011)	.476	.216 (.130)	.098
Deadline pressure^a^	−.017 (.051)	.745	−.017 (.051)	.747	−.007 (.042)	.865	−.013 (.015)	.383	−.270 (.169)	.109
Daily work hours^a^	.004 (.012)	.746	.004 (.012)	.738	.001 (.010)	.912	.003 (.004)	.326	.016 (.040)	.691
Having a steady partner	−.286 (.043)	.000	−.303 (.043)	.000	−.260 (.035)	.000	−.032 (.012)	.008	−.898 (.136)	.000
Constant	1.483 (.126)	.000	1.001 (.154)	.000	.980 (.127)	.000	.057 (.044)	.192	−4.962 (.424)	.000
N of person-years	5,656		5,656		5,656		5,656		5,656	
N of respondents	1,044		1,044		1,044		1,044		1,044	

a Centered at the sample median and coded as zero for those without jobs

*Note*: Values in parentheses are standard errors. Unless otherwise noted, all models presented in the table are fixed-effects models. The models also include dummies for each survey round, educational level, whether respondents were in school, and residential location, but the coefficients are omitted to conserve space. Following *Demographic Research*’s guidelines, we present p-values instead of symbols referring to discretized p-value intervals.

## Footnotes

3 Although there were some fluctuations, the follow-up rates for most waves are close to or above 80%.

4 Our exploratory analysis indicated that respondents who had chosen the response “never thought about marriage” are disproportionately more likely than others to select “would be fine either marry or not marry” at a later round, suggesting an affinity between the two reported views. The results also did not change in any meaningful way when we excluded observations reporting “never thought about marriage” (9.5%) or considered them as a separate category in an additional analysis.

5 An arranged date is a modern version of an arranged marriage, with the arranged couple being given the freedom to date for a while and decide whether to proceed to marriage.

6 For all but one job attribute, we found that the majority of respondents have experienced some variation in their reported conditions. For measures about job-specific conditions, 80–95% of respondents reported different conditions over time. The percentage reporting any variation is over 60% even for most measures of workplace characteristics.

7 In an earlier analysis we also included a dummy variable for observations in which we imputed income values. We found this variable to be hardly relevant in all models and the other results were virtually unchanged. For simplicity, we omit the dummy for imputed income in the presented models.

8 The only exception is income, because respondents can report income for the past year even without current jobs.

9 This is because after the transformation of the job trait variables, a person with median job traits would have the value 0 for all the traits, identical to one without a job, but the former is coded as 1 and the latter 0 for the indicator of having a job. Coding job characteristics as 0 or any other value for the jobless does not matter because the contribution of job characteristics to the outcome variable is conditional on having a job; the multiplication of having a job (= 0) with any assigned values for job characteristics will be 0 when respondents have no job.

10 Including both school enrollment and work status does not cause the problem of multicollinearity because a few respondents in the sample reported to be in school and hold jobs at the same time. We should nevertheless note that because we rely exclusively on within-person variations to estimate the models, education and school enrollment only capture *changes* in the educational level and enrollment status for each individual during the observed period. As such changes are rare among Japanese people in their late 20s and older, our ability to estimate the effects of education and school enrollment may be hampered by the rarity of within-person variations in these predictors in our sample. Removing these controls, however, did not substantively alter the results in an earlier analysis.

11 Because the measure of marital intention is derived from ordinal response categories, we also fitted fixed-effects ordered logit models, with the “blow up and cluster” estimator introduced by Baetschmann, Staub, and Winkelmann (2015), instead of the linear fixed-effects models specified here, in a separate analysis. We found the results to be highly similar.

12 The survey-year fixed effects also capture all the changes in individuals’ age between surveys, making it unnecessary to control for age.

13 About 70% of the women and 80% of the men in our sample showed no across-wave variation in the use of a formal method to seek partners. The small variation is unique to this outcome variable, because relatively few singles participate in any formal partner-seeking activity and because the variable is binary. Despite the severe sample reduction, our results generally held when we used fixed-effects logit models in an exploratory analysis.

14 When we compared the differences with zero, the p-values were smaller than 0.05.
